# The CogBIAS longitudinal study of adolescence: cohort profile and stability and change in measures across three waves

**DOI:** 10.1186/s40359-019-0342-8

**Published:** 2019-11-15

**Authors:** Charlotte Booth, Annabel Songco, Sam Parsons, Lauren Charlotte Heathcote, Elaine Fox

**Affiliations:** 1 Department of Experimental Psychology, University of Oxford, Anna Watts Building Radcliffe Observatory Quarte, Woodstock Road, Oxford, OX2 6GG UK; 2 Department of Anesthesiology, Perioperative, and Pain Medicine, Stanford University School of Medicine, 1070 Arastradero Road, Palo Alto, CA 94304 USA

**Keywords:** Cognitive, Behavioural, Mood, Impulsivity, Longitudinal, Stability, Change, Adolescent, Development

## Abstract

**Background:**

Adolescence is a time of considerable social, cognitive, and physiological development. It reflects a period of heightened risk for the onset of mental health problems, as well as heightened opportunity for flourishing and resilience. The CogBIAS Longitudinal Study (CogBIAS-L-S) aims to investigate psychological development during adolescence.

**Methods:**

We present the cohort profile of the sample (*N* = 504) across three waves of data collection, when participants were approximately 13, 14.5, and 16 years of age. Further, we present descriptive statistics for all of the psychological variables assessed including (a) the self-report mood measures, (b) the other self-report measures, and (c) the behavioural measures. Differential and normative stability were investigated for each variable, in order to assess (i) measurement reliability (internal consistency), (ii) the stability of individual differences (intra-class correlations), and (iii) whether any adolescent-typical developmental changes occurred (multilevel growth curve models).

**Results:**

Measurement reliability was good for the self-report measures (> .70), but lower for the behavioural measures (between .00 and .78). Differential stability was substantial, as individual differences were largely maintained across waves. Although, stability was lower for the behavioural measures. Some adolescent-typical normative changes were observed, reflected by (i) worsening mood, (ii) increasing impulsivity, and (iii) improvements in executive functions.

**Conclusions:**

The stability of individual differences was substantial across most variables, supporting classical test theory. Some normative changes were observed that reflected adolescent-typical development. Although, normative changes were relatively small compared to the stability of individual differences. The development of stable psychological characteristics during this period highlights a potential intervention window in early adolescence.

## Background

Adolescence is a period that entails significant social, cognitive, and physiological development. It reflects a period of protracted neurodevelopment, contributing to sensitivity towards the development of mental health problems, as well as adaptive and resilient outcomes [1, 2]. Many mental health problems, including anxiety, depression and substance use disorders, have their onset in adolescence, with prevalence rates steadily increasing throughout this period [3]. In 2017, it was estimated that 14% of UK secondary school children (aged 11 to 16) were living with a diagnosable mental health condition [4], which reflected an increase from previous reports [5]. Less research has investigated resilient outcomes in adolescence, despite that many individuals appear to maintain a good level of psychological wellbeing during this period. More longitudinal research is needed to track mental health development in normative adolescent samples, in order to identify early risk and protective factors for mental health problems and to define markers of resilience and wellbeing. The CogBIAS Longitudinal Study (CogBIAS-L-S) collected psychological data from a normative UK sample of adolescents (*N* = 504), at three time points across secondary school. The current paper is descriptive in nature, presenting the cohort profile and descriptive statistics for all of the psychological variables assessed. Predictive associations between specific variables will be addressed in future papers.

Theories of adolescent development are rooted within a biopsychosocial framework. The brain undergoes protracted development during adolescence, reflected by cortical thinning and myelin synthesis throughout many regions [6]. Neurodevelopmental changes are thought to occur non-linearly, with particular protracted maturation of prefrontal regions, in comparison to subcortical limbic systems [2]. This dual-systems developmental model has been linked to adolescent-typical behaviour, such as increasing levels of impulsivity and risk-taking [7, 8]. Changes in the limbic system have been linked to altered decision-making, heightened emotional responding and increased risk-taking, while protracted myelin synthesis in the pre-frontal cortex has been linked to improvements in executive functions [9]. Executive functions, such as attention control, cognitive flexibility, and information processing, show considerable improvement throughout childhood and adolescence, peaking at around 15 years of age [10, 11]. Adolescence is also characterised by changes in environmental processing, as adolescents become more susceptible to social input [12]. For example, early adolescents (aged 12 to 14 years) have been shown to be more socially influenced by their peers than by adults [13]. This effect is not typically found in any other age group, including older adolescents (aged 15 to 18 years), suggesting that young adolescents are particularly influenced by their peers. These factors contribute to the understanding of adolescence as a period of increased prosocial, as well as antisocial behaviour [14].

Adolescence also reflects a period of substantial emotional development. Adolescents are at increased risk for developing mood disorders, which has been linked to heightened levels of emotional reactivity and stress [15]. Many social, cognitive, and physiological changes that take place during the secondary school period may contribute to this increased risk. More longitudinal research is needed during this period of development, to provide a better understanding of early risk and protective factors. Environmental risk factors have previously been implicated, such as peer victimisation, family discord, and stressful life events [16–18]. There are also likely to be multiple genes contributing to the onset of mood disorders, which are thought to interact with environmental factors to increase risk [19]. Recent theories of adolescent mood disorder have implicated certain cognitive styles and information processing biases as mediating mechanisms in this risk model [20, 21]. Cognitive factors, such as worry, rumination, self-esteem, and information-processing biases in attention, interpretation and memory have been suggested as important factors [22–24]. Most of these factors can be described as continuous bi-polar constructs, providing either risk or protective mechanisms at either end of the continuum. These factors are also regarded as transdiagnostic, as they have been shown to predict both anxiety and depression outcomes [20]. While previous studies have shed light on risk and protective factors, more research is needed using longitudinal designs, in order to provide a better understanding of how these mechanisms develop and work together to influence mental health during adolescence.

The primary aim of CogBIAS-L-S is to investigate risk and protective factors underlying emotional vulnerability and resilience in adolescence. A wide range of self-report and complementary behavioural measures were assessed at three time points. Many mood-related variables were assessed, including symptoms of anxiety and depression, worry and rumination, as well as information-processing biases in attention, interpretation, and memory. A secondary aim is to investigate the development of executive functions and impulsivity-related behaviour, including risk-taking and overeating, in order to provide a more comprehensive understanding of how these behaviours develop during adolescence. Sensitivity to food cues has been used to test cognitive models of reward processing, therefore bias to approach food was investigated, together with relevant self-report measures [25, 26]. A tertiary aim is to investigate the role of cognitive biases in the development of pain-related distress. Chronic pain impacts a quarter of young people [27], follows a similar developmental trajectory as anxiety, and cognitive biases have been implicated in its development [28].

A three-wave longitudinal design was used, in order to provide a model for testing individual and sample level developmental change. Over 500 adolescents were recruited from UK secondary schools and completed the same battery of measures at each wave. Participants were first assessed near the beginning of starting secondary school and were followed for 4 years, completing the same measures every 12 to 18 months. This design was based on feasibility, in order to provide enough data to examine longitudinal stability and change across this developmental period. Saliva samples were collected at baseline and genome-wide analysis was conducted, although will be reported elsewhere. The in-depth assessment of mood and impulsivity-related variables across three waves, together with genome-wide data, provides a rich and unique dataset for examining risk and protective pathways in adolescence.

In this paper, we present the cohort profile and preliminary data on stability and change in the psychological variables assessed. Our aims were threefold: (i) to assess the reliability of the battery of measures, (ii) to assess the stability of each variable across waves, and (iii) to assess whether any adolescent-typical change was observed for each variable. Descriptive statistics are presented across the sample for: (a) the self-report mood measures, (b) the other self-report measures, and (c) the behavioural measures. Stability and change in the variables was investigated with multiple methods. Measurement reliability was assessed by checking internal consistency, in order to provide support for any evidence of stability and change observed. Differential (or rank-order) stability refers to whether individual differences are maintained over time, which was assessed using inter-wave reliability estimates [29]. Normative stability refers to whether change occurs at the sample level, which was assessed using multilevel growth curve analyses [29, 30]. Together, these methods provide a comprehensive investigation into stability and change.

We expected to observe substantial differential stability, such that individual differences would be maintained across waves. This is in line with classical test theory, which posits that psychological characteristics are stable across time, assuming high levels of measurement reliability [31]. However, we are investigating a particularly transient developmental period, therefore we expected to observe some adolescent-typical changes across the sample. In particular, we anticipated to observe worsening mood outcomes, increasing levels of impulsivity-related behaviour, as well as improvements in executive functions. Overall, we expected that differential stability would supersede normative stability, reflecting the relative strength of stability in individual differences over time.

## Method

### Participants

Participants were 504 secondary school children, sampled from nine different schools in the South of England. There were 10 different cohorts in the sample, as one school entered two consecutive year groups into the study. Twenty percent of the schools that were contacted agreed to participate. Students from an entire year group, near the beginning of their secondary school education (Years 7–9), were invited to take part. The range in school years was due to the different school types, as some started secondary school later, which is common in private schools in the UK. Parental consent and adolescent assent was received for all participants. Participants were followed up over 4 years, completing testing on three separate occasions, spaced approximately 12 to 18 months apart.

For the total sample at Wave 1, mean age was 13.4 (*SD* = 0.7), 55% were female, and 75% were Caucasian. We observed an 11% drop-out rate at Wave 2 (*N* = 450), and a 19% drop-out rate at Wave 3 (*N* = 411). For the participants retained at Wave 2, mean age was 14.5 (*SD* = 0.6), 56% were female, and 76% were Caucasian. For the participants retained at Wave 3, mean age was 15.7 (*SD* = 0.6), 58% were female, and 76% were Caucasian. We inferred level of Socio-economic Status (SES) from an average score for their parent’s highest level of education (1 = “Secondary school”, 2 = “Vocational/technical school”, 3 = “Some college”, 4 = “Bachelor’s degree”, 5 = “Master’s degree”, 6 = “Doctoral degree”). Parental education has been shown to be a reliable indicator of SES, as education affects both income and occupation, whilst also being a source of parent’s values and communicative styles [32, 33]. Across the sample, the median level of parental education was 4 (*Interquartile Range* = 2). Table 1 presents the sample demographics by each wave and testing cohort. Differences between the sample retained and lost were explored with independent samples *t-*tests at Wave 1 and Wave 3. Age, SES, cohort and ethnicity had no effect on whether participants were retained or lost. Gender did have an effect, *t* (502) = − 2.86, *p* = .004, *d* = .25, as more female participants were retained.*.*

**Table 1:** Sample demographics by each cohort group and wave

Wave 1											
Cohort	Total	X1	X2	X3	X4	X5	X6	X7	X8	X9	X10
*N*	504	15	30	62	47	13	34	119	104	54	26
*Mean Age (SD)*	13.4 (.7)	12.6 (.4)	11.7 (.3)	13.4 (.3)	13.4 (.3)	12.2 (.4)	12.8 (.3)	14.0 (.4)	13.1 (.3)	14.3 (.3)	13.2 (.3)
*Year group*	7–9	7–8	7	8	8	7–8	8	9	8	9	8
*Gender (% Female)*	55%	40%	50%	100%	100%	100%	47%	0%	100%	0%	58%
*Ethnicity (% Caucasian)*	75%	60%	87%	68%	72%	69%	59%	86%	69%	76%	85%
*SES (Median, IQR)*	4 (2)	4 (2)	3 (2)	4 (2)	3 (2)	4 (2)	2 (2)	4 (1)	4 (2)	4 (2)	3 (2)
Wave 2											
Cohort	Total	X1	X2	X3	X4	X5	X6	X7	X8	X9	X10
*N*	450	9	25	60	40	6	26	109	101	50	24
*Mean Age (SD)*	14.5 (.6)	14.0 (.4)	13.3 (.3)	14.5 (.3)	14.8 (.3)	13.5 (.2)	14.0 (.3)	15.1 (.4)	14.1 (.3)	15.4 (.3)	14.3 (.3)
*Year group*	8–10	8–9	9	9	10	8–9	9	10	9	10	9
*Gender (% Female)*	56%	56%	52%	100%	100%	100%	42%	0%	100%	0%	58%
*Ethnicity (% Caucasian)*	75%	56%	84%	67%	73%	67%	65%	86%	69%	74%	42%
*SES (Median, IQR)*	4 (2)	4 (2)	3 (2)	4 (2)	3 (2)	4 (2)	2 (2)	4 (1)	4 (2)	4 (2)	3 (2)
Wave 3											
Cohort	Total	X1	X2	X3	X4	X5	X6	X7	X8	X9	X10
*N*	411	8	22	62	37	12	12	92	92	50	24
*Mean Age (SD)*	15.7 (.6)	15.3 (.4)	14.8 (.3)	15.9 (.3)	15.8 (.3)	14.5 (.4)	15.0 (.3)	16.0 (.4)	15.4 (.3)	16.1 (.3)	15.3 (.3)
*Year group*	9–11	10–11	10	11	11	9–10	11	11	11	11	10
*Gender (% Female)*	58%	50%	46%	100%	100%	100%	67%	0%	100%	0%	58%
*Ethnicity (% Caucasian)*	76%	63%	86%	68%	73%	75%	75%	85%	70%	74%	88%
*SES (Median, IQR)*	4 (2)	4 (2)	3 (2)	4 (2)	3(2)	4 (2)	2 (2)	4 (1)	4 (2)	4 (2)	3 (2)

**Note:** Update from the protocol paper (Booth et al., 2017); age has now been coded to two decimal places, and SES (Socio-Economic Status) is the median of both mother and father education level; *SD* Standard Deviation; *IQR* Interquartile Range; 11% attrition at Wave 2 and 19% attrition by Wave 3

### Measures

#### Self-report mood measures

**Anxiety and Depression** was measured with the *Revised Child Anxiety and Depression Scale short form* (RCADS-SF) [34]. The scale consists of 25 items of internalising symptoms. Respondents are asked to indicate how often each item happens to them using a 4-point scale ranging from 0 (“Never”) to 3 (“Always”). Depression was assessed with 10 items (e.g., “I feel sad or empty”, “Nothing is much fun anymore”) and Anxiety was assessed with 15 items (e.g., “I feel scared if I have to sleep on my own”, “I worry that something bad will happen to me”). Anxiety can be further broken down using subscales for Social Anxiety, Separation Anxiety, General Anxiety, Panic Disorder and Obsessive Compulsive Disorder (OCD), which are each assessed with 3 items. Item responses were summed for Anxiety and Depression, with high scores reflecting greater internalising symptoms. For the Anxiety subscales, item responses were mean score averaged, with high numbers reflecting greater anxiety symptoms.

**Resilience** was measured with the *Connor-Davidson Resilience Scale short form* (CDR-SF) [35]. The scale consists of 10 items designed to measure trait resilience (e.g., “I believe I can achieve my goals even if there are obstacles”). Respondents are asked to think back over the past month and indicate whether each item applies to them, using a 5-point scale ranging from 0 (“Not true at all”) to 4 (“True nearly all the time”). Items responses were summed, with high scores indicating greater Resilience.

**Wellbeing** was measured with the *Mental Health Continuum short form* (MHC-SF) [36]. Respondents are asked to indicate how often they have experienced each of 14 different items over the past month (e.g., “happy”, “interested in life”), using a 6-point scale ranging from 0 (“Never”) to (“Every day”). Wellbeing can be further broken down using emotional, social and psychological subscales, although these are not reported in the present analyses. Item responses were summed, with high scores indicating greater Wellbeing.

**Self-esteem** was measured with the *Rosenberg Self-Esteem scale* (RSE) [37]. The scale consists of 10 items measuring self-worth and acceptance (e.g., “I feel that I have a number of good qualities”, “On the whole I am satisfied with myself”). Respondents are asked to indicate how much they agree with each item using a 4-point scale ranging from 0 (“Strongly disagree”) to 3 (“Strongly agree”). Item responses were averaged, with high scores indicating better Self-esteem.

**Worry** was measured with the *Penn State Worry Questionnaire for Children* (PSWQ-C) [38]. The scale consists of 14 items designed to measure the tendency to worry in children aged 6 to 18 years old. Respondents are asked to indicate how true each item is for them (e.g., “My worries really worry me”, “I know I shouldn’t worry, but I just can’t help it”), using a 4-point scale ranging from 0 (“Never true”) to 3 (“Always true”). Item responses were averaged, with high scores reflecting a greater tendency to Worry.

**Rumination** was measured with the *Children’s Response Style Scales* (CRSS) [39]. This scale measures both Rumination (negative) and Distraction (positive), which are cognitive styles that present in response to adverse experiences. The Rumination scale consists of 10 items (e.g., “When I feel sad, I think back to other times I have felt this way”) and the Distraction scale also consists of 10 items (e.g., “When I feel sad, I think about something I did a little while ago that was a lot of fun”). Respondents are asked to indicate how true each item is for them using an 11-point scale ranging from 0 (“Never”) to 10 (“Always”). Item responses for each scale were averaged, with high scores reflecting a greater tendency towards Rumination and Distraction respectively.

#### Other self-report measures

**Life events** were measured with the *Child Adolescent Survey of Experiences* (CASE) [40]. The survey consists of 38 life events, relevant to children and adolescents (e.g., “My parents split up”, “I went on a special holiday”). Respondents are asked to indicate whether each particular event happened to them during the past 12 months, and if so, they are asked to rate the event using a 6-point scale (1 = “Really bad”, 2 = “Quite bad”, 3 = “A little bad”, 4 = “A little good”, 5 = “Quite good”, 6 = “Really good”). They are also given the option to include a further two life events, which they are asked to rate using the same scale. A score for Positive Life Events was computed as the number of events experienced and rated as either really good, quite good, or a little good by the respondent. A score for Negative Life Events was computed as the number of events experienced and rated as really bad, quite bad, or a little bad by the respondent.

**Victimisation** was measured with the *Multidimensional Peer Victimisation Scale* (MPVS) [41]. The scale consists of 16 items relating to bullying perpetrated by peers (e.g., “Beat me up”, “Swore at me”, “Tried to make friends turn against me”). Respondents are asked to indicate how often each item happened to them in the past 12 months using a 3-point scale (0 = “Not at all”, 1 = “Once”, 2 = “More than once”). Subscales can be calculated referring to physical, verbal, social and property vandalism, although for the current paper, only the total score was examined. Item responses were summed to create the total score, with high scores indicating greater levels of Victimisation.

**Impulsivity** was measured with the *UPPS Revised Child version* (UPPS-R-C) [42]. It is a 32-item questionnaire measuring Lack of Premeditation (e.g., “I tend to blurt things out without thinking”), Negative Urgency (e.g., “When I feel bad, I often do things I later regret in order to feel better now”), Sensation Seeking (e.g., “I would enjoy water skiing”) and Lack of Perseverance (e.g., “I tend to get things done on time”- reverse scored”). Respondents are asked to indicate how much each item describes them personally using a 4-point scale ranging from 1 (“Not at all like me”) to 4 (“Very much like me”). Items corresponding to each subscale were averaged, with high numbers reflecting greater impulsivity.

**Behavioural Inhibition and Activation** (BIS/BAS) was measured with the *BIS/BAS Scales for Children* [43]. The scale consists of 20 items in total, corresponding to BIS (e.g., “I feel pretty upset when I think that someone is angry with me”), BAS-Drive (e.g., “I do everything to get the things that I want”), BAS-Reward Responsiveness (RR: e.g., “When I am doing well at something, I like to keep doing it”) and BAS-Fun Seeking (Fun: e.g., “I often do things for no other reason that they might be fun”). Respondents are asked to indicate how much they agree or disagree with each item using a 4-point scale (0 = “Not true”, 1 = “Somewhat true”, 2 = “True”, 3 = “Very true”). Items corresponding to each component were averaged, with high numbers reflecting greater agreement.

**Risk behaviour** was measured with a modified version of the *Risk Involvement and Perception Scales* (RIPS) [44]. We used 14 of the original 23 risky behaviours, which were deemed to be suitable for our younger UK sample, as the original scale was used in older American adolescents. Respondents were asked whether, during the past 12 months, they engaged in each of the risky behaviours (e.g., riding in a car without a seatbelt, drinking alcohol, skipping school). They were then asked to rate how bad they consider the consequences of each behaviour to be, followed by rating how good they consider the benefits of each behaviour to be, using a 9-point scale from 0 (“Not bad/good at all”) to 8 (“Really bad/good”). A score for Risk Involvement was computed as the sum of the frequency ratings. A score for Risk Perception and Benefit Perception was computed as the average of the item responses for these scales respectively.

**Overeating** was measured with the *Three-Factor Eating Questionnaire* (TFEQ-18) [45]. The scale consists of 18 items designed to measure three eating styles, which are Cognitive Restraint (e.g., “I consciously hold back at meals in order not to gain weight”), Uncontrolled Eating (e.g., “Sometimes when I start eating, I just can’t seem to stop”) and Emotional Eating (e.g., “When I feel blue, I often overeat”). Respondents are asked to rate how true each item is of them using a 4-point scale (0 = “Definitely false”, 1 = “Mostly false”, 2 = “Mostly true”, 3 = “Definitely true”). Scores for each subscale were computed by summing the relevant items, so that high scores indicated greater overeating.

**Pain Catastrophising** was measured with the *Pain Catastrophising Scale for Children* (PCS-C) [46]. The scale consists of 13 items designed to measure cognitions associated with the experience of pain (e.g., “When I’m in pain, I become afraid that the pain will get worse”, “When I’m in pain, I become afraid that the pain will get worse”). Respondents are asked to indicate how likely they are to have these thoughts when they are experiencing pain, using a 5-point scale ranging from 0 (“Not at all”) to 4 (“All the time”). Subscales can be computed for rumination, magnification and helplessness, although the current analyses were conducted on the total score. Item responses were summed, with high scores reflecting greater levels of Pain Catastrophising.

#### Behavioural measures

**Memory bias** was assessed with a Self-Referential Encoding Task (SRET). The task consisted of three phases: an encoding phase, a distraction phase, and a surprise recall phase. In the encoding phase, participants were shown 22 positive (e.g., “cheerful”, “attractive”, “funny”) and 22 negative (e.g., “scared”, “unhappy”, “boring”) self-referent adjectives one at a time, in a random order, and asked to indicate whether each word described them, by pressing the “Y” or “N” keys on the keyboard. The 44-item word list had been matched for length and recognisability in adolescents in a previous study [47]. In the distraction phase, participants were asked to complete three maths equations (e.g., “What is 2 x 3?”), one at a time, in a fixed order. Responses did not have to be correct and answers were not given. In the surprise recall phase, a large answer box was displayed on the screen and participants were asked to type as many words as they could remember, both good and bad, from the ‘Describes me?’ task. The phase ended after 3 mins. A score was computed for the number of negative words endorsed and recalled (Negative Recall), number of positive words endorsed and recalled (Positive Recall), as well as the total number of words endorsed and recalled (Total). Memory Bias was computed as: ((Negative Recall – Positive Recall) / Total). This created a score whereby 0 indicated no bias, negative scores indicated a more positive bias, and positive scores indicated a more negative bias. The bias score was computed in this way so that high numbers indicated increased risk for psychopathology.

**Interpretation bias** was measured with the *Adolescent Interpretation and Belief Questionnaire* (AIBQ) [48]. In this task, participants are asked to imagine themselves in 10 different ambiguous scenarios and following each one are asked to indicate how likely each of three possible interpretations would be to pop into their mind. Five scenarios are social and five are non-social in nature. An example of a social scenario is “You’ve invited a group of classmates to your birthday party, but a few have not yet said if they are coming”. Participants then rate how likely a negative (i.e., “They don’t want to come because they don’t like me”), positive (i.e., “They’re definitely coming; they don’t need to tell me that”) and neutral (i.e., “They don’t know if they can come or not”) interpretation is to pop into their mind using a 5-point scale (1 = “Doesn’t pop up in my mind”, 3 = Might pop up in my mind”, 5 = “Definitely pops up in my mind”). A forced choice question is shown following these ratings, asking which the most believable interpretation is, although this question is generally not used for analysis. A score for Positive Social, Negative Social, Positive Non-Social and Negative Non-Social was computed as the average of the respective items. Scores ranged from 1 to 5. A Social Interpretation Bias score (Negative Social – Positive Social) and a Non-Social Interpretation Bias score (Negative Non-Social – Positive Non-Social) was then computed in order to create a bias score, whereby higher scores indicated greater negative interpretations for social and non-social situations respectively.

**Attention bias** was measured with a pictorial Dot-Probe task [49]. The task consisted of three blocks, corresponding to the assessment of attentional biases to: (*i*) threat (i.e., angry faces), (*ii*) pain (i.e., pain faces), and (*iii*) positivity (i.e., happy faces). The faces were chosen from the STOIC faces database [50], which are images of faces presented in greyscale with no hair or jawline showing. Seven actors were used, eight times within each block. Pictures were 230 × 230 pixels in size, presented approximately 10 degrees visual angle apart. Each block consisted of 56 trials, whereby an emotional face was paired with a neutral face (of the same actor), displayed for 500 ms. This was followed by a probe, in the centre of the space previously occupied by one of the faces. Probes were letters ‘Z’ and ‘M’, and were displayed for 3000 ms, or until a response was made. Participants were instructed to respond to the probe as fast and accurately as possible, pressing the respective ‘Z’ or ‘M’ key on the keyboard. There was an inter-trial interval of 500 ms, followed by a fixation cross for 500 ms, indicating the start of a new trial. An error message was shown following an incorrect response or following no response (i.e., slower than 3000 ms). Block order was counterbalanced and trials within each block were randomised. A rest period of 30,000 ms was given between blocks, which was indicated by a countdown timer. Practice blocks were given first responding to only the probes (8 trials), then responding to the probes behind neutral-neutral face pairings (16 trials). In experimental trials, congruent trials refer to when the probe appears behind an emotional face, and incongruent trials refer to when the probe appears behind a neutral face. There were equal numbers of congruent and incongruent trials. As standard, a bias score was computed by subtracting mean RT for congruent trials from mean RT for incongruent trials. Positive bias scores are thought to indicate emotional vigilance and negative scores are thought to reflect emotional avoidance. Incorrect trials, fast (< 200 ms), slow (> 3000 ms), and extreme responses (3 *SD*s from each participant’s mean RT for each trial type/ emotion category respectively) were not analysed. Participants who made more than 30% errors overall were excluded. Indices were calculated for Angry Bias, Pain Bias and Happy Bias from the respective blocks.

**Risk-taking** was assessed with the *Balloon Analogue Risk Task for Youth* (BART-Y) [51]. The script was a modified version of the BART-Y downloaded from the Inquisit Test Library, as less trials were shown. In this task, participants are instructed to pump a computer-generated red balloon using a button displayed below the balloon, and to ‘bank’ the points gained from each pump, using a different button displayed below a points meter. Each balloon press gains one point and the aim of the task is to bank as many points as possible. Participants were instructed that balloons can burst at any point and that they should bank their points before they think the balloon will burst. Responses were made with the left mouse button. The balloon pump button caused the balloon to either increase in size or to burst, and the points meter button caused the points meter to increase. If a balloon burst, then no points were won on that trial and a new trial started. Twenty trials were completed, which was less than the original study, due to time constraints of our study design. For each trial, the average bursting point was 60 pumps, which ranged from 10 to 111 pumps. The average number of pumps on the balloons that did not burst was used as an index of risk-taking.

**Cognitive interference** was assessed with a *Flanker Task* [52]. The script was a modified version of the ‘Child Flanker Test (with fish)’ downloaded from the Inquisit Test Library. The task differs from the adult version, as pictures of fish are used instead of arrows. Stimuli were yellow fish embedded with a faint black arrow (150 × 230 pixels). Participants are instructed to indicate whether a fish displayed in the centre of the screen is pointing either left or right, whilst ignoring two flanker fish on either side of the target fish. Flankers either point in the same direction as the target fish (i.e., congruent trials), or point in the opposite direction as the target fish (i.e., incongruent trials), which cause interference. Four trial types: target point left (congruent); target point right (congruent); target point left (incongruent); target point right (incongruent); were displayed 29 times each in random order. A rest period of 30,000 ms was given halfway through the task, which was indicated by a countdown timer. Participants were instructed to respond to the target as fast and accurately as possible. Incorrect trials, fast (< 200 ms), slow (> 3000 ms), or extreme responses (3 *SD*s from each participant’s mean RT for each condition) were not analysed. Flanker Interference was computed by subtracting mean RT for congruent trials from mean RT for incongruent trials. High scores indicate more interference, therefore poor attention control.

**Food Approach bias** was assessed with a Stimulus-Response Compatibility task [26]. The script was a modified version of the ‘Manikin Task’ downloaded from the Inquisit Test Library. The task consisted of two blocks: (*i*) a food approach/non-food avoid block, and (*ii*) a food avoid/non-food approach block – which were counterbalanced in order of presentation. Participants were instructed to either approach or avoid each stimulus type at the beginning of the block. A trial began with a fixation cross in the centre of the screen (1000 ms), replaced by a stimulus (food or non-food picture) in the centre of the screen with a manikin (15 mm high) positioned 40 mm above or below the picture. There was a brief inter-trial interval (500 ms). The task consisted of 112 experimental trials (approach food, avoid food, approach non-food, and avoid non-food trials in equal number). Approach and avoidance responses were made by pressing the up or down arrow keys. Responding caused the manikin to become animated and move in the direction of the arrow press. Each trial was completed when the participant had made three responses and the manikin had either reached the picture (approach trials) or reached the top/bottom of the screen (avoid trials). Only the initial RT was used for data analysis. Pictures were chosen from the food-pics database [53], which contains over 800 images of food and non-food items, rated on perceptual characteristics and affective ratings. We chose 8 sweet snack food pictures (e.g., donut, ice-cream, grapes and blueberries) and 8 non-food miscellaneous household pictures (e.g., cushion, key, book and umbrella) that were matched for complexity, familiarity and valence. Incorrect responses, fast (< 200 ms), slow (> 3000 ms), and extreme responses (3 *SD*s from each participant’s mean RT by block) were not analysed. Further, participants who committed more than 40% errors were excluded. A food bias score was calculated by subtracting the mean RT in the food approach/non-food avoid block, from the mean RT in the food avoid/non-food approach block, so that high scores indicated a stronger Food Approach Bias.

#### Body-mass index (BMI)

**Body-Mass Index** (BMI) was calculated (BMI: kg/m^2^) from measuring participant’s height (meters) and weight (kilograms) at each of the three waves using a Seca portable height measure and Salter portable weight scales.

#### Further measures added in wave 2

**Attention control** was measured with the Attentional Control Scale (ACS) [54]. The scale consists of 20 items related to the ability to focus and shift attentional resources (e.g., “It is hard for me to concentrate of a difficult task when there are noises around” – reverse scored, “I can quickly shift from one task to another”). Respondents are asked to indicate how each item relates to them using a 4-point scale (1 = “Almost never”, 2 = “Sometimes”, 3 = “Often”, 4 = “Always”). A score was computed by averaging the items, with high scores reflecting good attention control.

**Sensory-Processing Sensitivity** (SPS) was measured with the Highly Sensitive Child Scale (HSCS) [55]. The scale consists of 12 items (e.g., “Loud noises make me feel uncomfortable”, “Some music can make me really happy”). Respondents are asked to indicate how they feel personally about each item using a 7-point scale from 1 (“Not at all”) to 7 (“Extremely”). A score was computed by averaging all of the items, with high scores reflecting high SPS.

**Binge eating** was assessed in line with previous studies [56]. Participants were asked whether they had experienced an eating binge during the past month (0 = “Never”, 1 = “Less than once a month”, 2 = “1 to 3 times a month”, 3 = “Once a week”, 4 = “More than once a week”). They were then asked five more questions about whether they felt out of control during these episodes, as if they could not stop eating even if they wanted to, using a 3-point scale (0 = “No”, 1 = “Sometimes”, 2 = “Always”). Binge eating was coded as positive if they scored above 1 on both of these questions. This measured thus reflected a categorical outcome.

**Working memory** was assessed with the *Corsi-Block Tapping Task* (CBTT) [57]. Both the forward and backward CBTT were assessed. The scripts were downloaded from the Inquisit Test Library. In this task, nine blue squares are displayed on the screen (black background) in a pseudorandom position. The squares light up (change to yellow for 1 sec) in different sequences. In the forward task, participants are instructed to recall the sequence and click on the squares in the order they lit up. In the backward task, participants are instructed to recall the sequence backwards and click on the squares in the reverse order they lit up. The squares also change to yellow when participants recall the sequence by clicking on the square. Participants were instructed to click the button labelled ‘Done’ when they had finished recalling the sequence, or press the ‘Reset’ button if they made a mistake. The sequence length started at 2 and increased by 1 every time two sequences were recalled correctly. The task ended when participants recalled twice incorrectly. The maximum sequence length was 9. As standard, a score was computed by multiplying the highest achieved block span with the number of correctly recalled sequences. High scores indicate better working memory.

### Procedure

Schools were recruited by sending emails to head teachers or heads of psychology departments. Following this, an initial meeting with teachers was arranged, whereby the study commitment was explained in more detail and testing procedures were arranged. Parental consent forms were sent out to entire year groups of students either in paper format, or electronically, depending on the school’s preference. Parents were asked to read the information sheet and return the completed consent form and family demographic questionnaire, either to the school or directly to the research team. Test sessions were arranged during school hours, usually in computer rooms at the school, although two nearby schools came into the University of Oxford computer labs for testing. Adolescent assent forms were completed just before the initial test session, after they had read the adolescent information sheet and the study procedure had been verbally explained to them.

Test sessions lasted 2 hrs. This was either completed all at once, or on different days, as the sessions were split into shorter one-hour sessions. Each test session involved completing some behavioural tasks, programmed and delivered through Inquisit [58], followed by completing a batch of questionnaires, programmed and delivered through Limesurvey [59]. Testing was completed in groups, which ranged in size from 6 to up to 50 participants, depending on the size of the cohort and the available testing space. Participants were asked to read and follow the instructions for each task and questionnaire on the computer screen. At least two trained research assistants were always present to answer any questions. Participants were instructed to work in exam conditions throughout the session, which meant not talking or looking at their peers computer screen. Teachers from the school were also present to support test sessions. At the end of each wave of data collection, participants were thanked, debriefed and given a £10 Amazon voucher.

### Data analysis

The data was stored and preliminary analyses were conducted in SPSS [60]. We report descriptive statistics for each variable by their Mean (*M*) and Standard Deviation (*SD*). Internal consistency was calculated using coefficient omega for self-report variables and using split-half estimates for behavioural Reaction-Time (RT) based measures. We refer to internal consistency estimates > .70 as showing a high level of reliability. Coefficient omega has been described as a superior alternative to the widely used coefficient alpha, which holds highly stringent assumptions [61]. Omega was calculated using the free software JASP [62]. For RT variables, we report permutation based Spearman-Brown corrected split-half reliability, which was conducted using the ‘*splithalf*’ package [63] in *R* [64]. This procedure splits the data into two random halves (following the data reduction steps described above), calculating the difference score (i.e., bias score; incongruent minus congruent trials), and calculating the correlation between both halves (corrected with the Spearman-Brown prophecy formula). This procedure was repeated across 5000 permutations and we report the mean split-half reliability across all splits. This procedure is more robust than taking a single split (e.g., comparing first and last halves of trials, or comparing odd and even trials) to estimate internal consistency.

In order to assess differential stability, we examined inter-wave variability. The third form of the Intraclass Correlation Coefficient (ICC_3,1_), as described by Shrout and Fleiss [65], was calculated for each variable, estimating the correlation of measures across waves. The ICC was modelled by a two-way mixed effects model; random participant effects and fixed sessions effects, with absolute agreement. Higher values indicate higher stability across waves. We refer to ICC estimates > .70 as reaching a high level of stability [66].

To assess normative stability, we tested linear growth curve models using the ‘*nlme*’ package [67], in R [64], with Full Maximum Likelihood Estimation (FIML). Growth models were only tested if variables showed high stability across three waves. Missing data was treated as ‘missing at random’, so that participants who only took part only at Wave 1 could still contribute to the model estimates. A Multi-Level Model (MLM) framework was applied, as longitudinal data is considered nested (or dependent) on multiple assessments per each individual. Level-1 refers to the repeated measures of data nested in individuals and level-2 refers to the individual. Waves were coded as 0, 1 and 2, to set a baseline for the intercept [68]. The ratio of between-cluster variance to the total variance in each variable was assessed using the ICC from the intercept only model. Levels of ICC > .10 suggest that substantial clustering is taking place, which justifies using MLM over normal regression techniques [30]. After running an intercepts only model, a fixed slopes model was run, whereby the effect of wave was included. After this, a random slopes model was run, allowing intercepts and slopes to vary by individual. Deviance statistics were tested to compare model fit between the intercept only, fixed slopes, and random slopes models, using log-likelihood statistics. The average slope estimate (γ_10_) from the best fitting model indicated whether any significant change occurred across the sample. We used an adjusted significance level of *p* < .005, to correct for the large number of models tested. We did not include any time constant or time varying covariates to the models, as we aimed to focus purely on stability and change within each variable.

## Results

### Internal consistency

Internal consistency was first examined, using the omega coefficient (Mcdonald’s *ω*) for self-report measures and split-half estimates for the RT measures. Results are presented in Table 2. Bold indicates that the measure reached a high level of internal consistency. All of the mood and other self-report measures reached a high level of internal consistency, with the exception of the Separation Anxiety subscale from RCADS-SF. None of the behavioural measures reached a high level of internal consistency, apart from the Negative Social subscale from the AIBQ. Internal consistency was extremely low for the Dot-Probe variables (i.e., Angry, Happy, and Pain Bias), as these variables mostly did not reach statistical significance. However, internal consistency for the Dot-Probe variables did increase by wave, with the highest estimate reaching .27 for Angry Bias at Wave 3.

**Table 2:** Descriptive statistics, internal consistency (McDonald’s *ω* or split-half for RT measures), and differential stability (ICC) for all variables across waves

	Wave 1 (*N* = 504)			Wave 2 (*N* = 450)			Wave 3 (*N* = 411)			Differential stability		
*Self-report mood measures*												
	*M*	*SD*	*ω*	*M*	*SD*	*ω*	*M*	*SD*	*ω*	*ICC*	*CI Lower*	*CI Upper*
Anxiety	13.40	7.66	**.87**	14.28	7.92	**.87**	13.87	7.82	**.87**	**.82**	.79	.85
- Separation Anxiety	1.49	1.67	.64	1.50	1.82	.67	1.48	1.66	.59	**.78**	.74	.81
- Social Phobia	4.81	2.19	**.74**	5.37	2.23	**.76**	5.20	2.21	**.76**	**.77**	.72	.81
- Generalised Anxiety	3.36	2.25	**.75**	3.56	2.30	**.74**	3.43	2.25	**.72**	**.79**	.75	.82
- Panic Disorder	1.66	1.93	**.70**	1.84	2.07	**.73**	1.93	2.17	**.73**	**.75**	.71	.79
- O C D	2.09	2.11	**.70**	2.02	2.14	**.71**	1.83	2.28	**.83**	**.77**	.73	.81
Depression	8.25	5.39	**.87**	9.44	6.14	**.89**	10.18	6.34	**.90**	**.82**	.78	.85
Distraction	5.01	2.47	**.92**	4.86	2.55	**.94**	4.63	2.39	**.94**	.68	.61	.73
Rumination	5.70	2.13	**.88**	6.31	1.96	**.88**	6.53	1.89	**.89**	**.70**	.64	.76
Resilience	24.64	7.43	**.89**	23.33	7.68	**.89**	23.85	7.50	**.90**	**.80**	.76	.83
Self-esteem	1.83	.51	**.87**	1.78	.54	**.89**	1.71	.58	**.90**	**.80**	.77	.84
Wellbeing	40.75	12.58	**.94**	43.63	15.09	**.94**	41.61	15.74	**.95**	**.80**	.76	.84
Worry	1.55	.65	**.92**	1.67	.69	**.86**	1.65	.65	**.91**	**.84**	.81	.87
**Note**: *O C D* Obsessive Compulsive Disorder; Bold indicates high level of reliability/stability												
*Self-report other measures*												
	*M*	*SD*	*ω*	*M*	*SD*	*ω*	*M*	*SD*	*ω*	*ICC*	*CI Lower*	*CI Upper*
BIS	1.51	.54	**.73**	1.94	.49	**.73**	1.99	.51	**.73**	.64	.41	.76
BAS Drive	1.14	.68	**.78**	1.23	.67	**.80**	1.24	.67	**.81**	**.72**	.66	.77
BAS Fun	1.73	.64	**.70**	1.68	.66	**.80**	1.68	.65	**.72**	**.79**	.75	.83
BAS RR	2.17	.55	**.74**	2.03	.58	**.71**	2.03	.53	**.73**	.68	.61	.73
Cognitive Restraint	13.47	4.27	**.83**	13.33	4.49	**.86**	13.58	4.81	**.89**	**.73**	.68	.78
Emotional Eating	5.34	2.49	**.87**	6.06	2.77	**.89**	6.29	2.86	**.92**	**.72**	.66	.77
Uncontrolled Eating	19.63	5.83	**.88**	21.05	5.80	**.87**	20.95	5.69	**.86**	**.75**	.70	.79
Lack of Perseverance	2.17	.58	**.85**	2.07	.53	**.84**	2.04	.49	**.82**	**.74**	.68	.78
Lack of Premeditation	2.30	.55	**.83**	2.19	.50	**.81**	2.14	.49	**.82**	**.76**	.71	.80
Negative Urgency	2.47	.61	**.83**	2.55	.60	**.84**	2.56	.64	**.87**	**.79**	.75	.83
Sensation Seeking	2.99	.73	**.88**	2.92	.73	**.88**	2.91	.76	**.90**	**.91**	.89	.93
Negative Life Events	5.51	4.16	n/a	5.31	4.04	n/a	5.01	3.48	n/a	**.74**	.69	.78
Positive Life Events	6.89	3.37	n/a	6.35	3.36	n/a	6.09	3.02	n/a	.57	.49	.64
Pain Catastrophising	19.70	10.76	**.92**	19.69	10.83	**.92**	20.17	10.15	**.92**	.69	.63	.75
Risk Involvement	3.59	2.20	n/a	4.36	2.39	n/a	4.67	2.44	n/a	**.79**	.70	.85
- Risk Perception	5.52	1.28	**.87**	5.19	1.24	**.87**	4.99	1.17	**.86**	**.75**	.68	.81
- Benefit Perception	2.36	1.14	**.85**	2.75	1.18	**.86**	2.82	1.06	**.83**	**.76**	.69	.81
Victimisation	9.99	7.37	**.89**	10.16	7.72	**.91**	9.03	7.24	**.90**	**.79**	.75	.83
**Note:** *BIS* Behavioural inhibition; *BAS* Behavioural activation; *RR* Reward responsiveness; *n/a* Calculation was not appropriate for this data Bold indicates high level of reliability/stability												
Behavioural measures												
	*M*	*SD*	*ω*	*M*	*SD*	*ω*	*M*	*SD*	*ω*	*ICC*	*CI Lower*	*CI Upper*
BART	26.95	12.30	n/a	30.28	14.13	n/a	34.27	16.15	n/a	.60	.51	.68
BMI	19.89	3.27	n/a	20.69	3.18	n/a	21.24	3.20	n/a	**.94**	.87	.97
DP Angry bias (RT)	.60	43.32	−.02	2.50	30.62	.10	1.95	33.06	.27	.10	−.07	.25
DP Happy bias (RT)	3.07	44.99	.08	2.75	31.06	.18	−3.62	29.77	.22	−.03	−.22	.14
DP Pain bias (RT)	1.69	36.99	−.03	.44	29.99	.12	1.38	30.26	.16	−.12	−.34	.06
Flanker Interference (RT)	30.91	29.80	.42	26.63	22.38	.34	24.02	23.34	.40	.49	.39	.57
Food Bias (RT)	47.91	107.42	.67	58.15	122.89	**.72**	53.99	111.77	.69	.40	.29	.50
Memory Bias	−.49	.44	n/a	−.34	.43	n/a	−.30	.44	n/a	**.72**	.65	.77
- Negative Recall	2.39	2.29	n/a	3.59	2.70	n/a	3.93	2.89	n/a	.68	.59	.75
- Positive Recall	6.76	2.84	n/a	6.99	3.00	n/a	7.07	3.12	n/a	**.72**	.67	.77
Non-Soc. Interpretation Bias	−.35	1.03	n/a	−.41	1.00	n/a	−.53	1.00	n/a	**.74**	.69	.79
- Negative Non-Social	3.17	.71	.56	3.09	.69	.54	3.07	.74	.57	**.74**	.69	.78
- Positive Non-Social	3.51	.64	.46	3.50	.69	.57	3.60	.67	.60	**.70**	.64	.75
Social Interpretation Bias	.69	1.20	n/a	.61	1.25	n/a	.56	1.25	n/a	**.77**	.73	.81
- Negative Social	3.26	.88	**.78**	3.13	.94	**.81**	3.11	.97	**.84**	**.77**	.73	.81
- Positive Social	2.57	.63	.56	2.52	.65	.55	2.55	.67	.64	.64	.57	.70
***Note:*** *BART* Balloon analogue risk task; *BMI* Body-Mass-Index; *DP* Dot-probe; *RT* Reaction-Time; *n/a* Calculation was not appropriate for this data; Bold indicates high level of reliability/stability												
*Added in Wave 2*												
				*M*	*SD*	*ω*	*M*	*SD*	*ω*	*ICC*	*CI Lower*	*CI Upper*
Attention Control	–	–	–	2.48	.41	.**83**	2.48	.41	**.84**	**.76**	.71	.81
Binge Eating	–	–	–	34%	n/a	n/a	37%	n/a	n/a	.60	.61	.68
Corsi-block Forward	–	–	–	57.63	20.68	n/a	63.20	22.76	n/a	.62	.53	.70
Corsi-block Back	–	–	–	50.97	14.19	n/a	53.39	14.81	n/a	.35	.21	.47
SPS	–	–	–	4.36	.89	**.81**	4.37	.86	**.79**	**.76**	.71	.81

**Note:** Binge eating was a categorical outcome, therefore percentage refers to frequency of those who scored positive for Binge eating; *SPS* Sensory-Processing Sensitivity; *n/a* Calculation was not appropriate for this data; Bold indicates high level of reliability/stability

### Differential stability

Differential stability was assessed by examining inter-wave variability (ICC_3,1_). Parameter estimates with lower and upper Confidence Intervals (CI) are presented in Table 2. Bold indicates whether each variable showed high stability. Most of the mood and other self-report measures showed high levels of differential stability. However, there were some exceptions, including Distraction, BIS, BAS-RR, Positive Life Events, and Pain Catastrophising. In terms of the behavioural measures, high levels of stability were observed for Memory Bias, Non-Social Interpretation Bias, and Social Interpretation Bias. None of the other behavioural measures showed high stability. In particular, the Dot-Probe variables (i.e., Angry, Happy, and Pain Bias) showed no stability (i.e., non-significant) across waves.

### Normative stability

Growth curve models were conducted to examine normative stability, i.e., whether any change occurred across the sample. Only variables that showed high stability were tested and subscales were not examined, to reduce the number of models tested. For the self-report mood measures these included: Anxiety, Depression, Rumination, Resilience, Self-esteem, Wellbeing, and Worry. For the other self-report measures these included: BAS Drive, BAS Fun, Cognitive Restraint, Emotional Eating, Uncontrolled Eating, Lack of Perseverance, Lack of Premeditation, Negative Urgency, Sensation Seeking, Negative Life Events, Risk Involvement, and Victimisation. For the behavioural measures these included: Memory Bias, Non-Social Interpretation Bias, and Social Interpretation Bias. To assess model fit, we compared the log-likelihood deviance (−2LL) between the intercept only, fixed slopes, and random slopes models. Parameter estimates are shown in Table 3, with the best fitting model shown in bold. The intercept (γ_00_) is the average score for the sample at baseline. Although, the intercept only model does not include the effect of wave, therefore γ_00_ here is the average score across all waves. The slope (γ_10_), or fixed effects, represent the average change in each variable, per each assessment wave. Due to the large number of models tested, we used an adjusted level of significance (at *p* < .005), to indicate significant change. Random effects are also depicted in Table 3, represented by (*i*) the intercept variance (τ_00_), (*ii*) the slope variance (τ_11_), and (*iii*) their covariance (τ_01_). All models show intercept variance, but only the random slopes model shows slope variance and covariance. Positive covariance suggests that a high score at baseline predicts an increasing score over time. Negative covariance suggests that a high score at baseline predicts a decreasing score over time.

**Table 3:** Parameter estimates for linear growth curve models testing change over time at three waves

Self-report mood measures		Model test				Fixed effects					Random effects		
		ICC	-2LL	*∆ p*	γ_00_	SE	*p*	γ_10_	SE	*p*	τ_00_	τ_11_	τ_01_
Anxiety	Intercept only	.60	− 4444.00		13.73	0.30	<.001				6.02		
*N* = 504	Fixed slopes		− 4443.34	.252	13.56	0.34	<.001	0.19	0.17	.252	6.02		
	Random slopes		**− 4427.29**	**<.001**	**13.56**	**0.34**	**<.001**	**0.21**	**0.19**	**.283**	**6.57**	**2.68**	**−.32**
Depression	Intercept only	.58	− 4099.91		9.14	0.23	<.001				4.53		
*N* = 504	Fixed slopes		− 4073.72	<.001	8.31	0.25	<.001	0.95	0.13	<.001	4.56		
	Random slopes		**− 4050.70**	**<.001**	**8.31**	**0.24**	**<.001**	**0.94**	**0.15**	**<.001**	**4.48**	**1.99**	**−.07**
Rumination	Intercept only	.47	− 2633.23		6.08	0.08	<.001				1.41		
*N* = 502	Fixed slopes		− 2607.49	<.001	5.76	0.09	<.001	0.38	0.05	<.001	1.41		
	Random slopes		**− 2593.13**	**<.001**	**5.75**	**0.09**	**<.001**	**0.39**	**0.06**	**<.001**	**1.73**	**0.74**	**−.56**
Resilience	Intercept only	.56	− 4236.59		23.95	0.29	<.001				5.65		
*N* = 500	Fixed slopes		**− 4234.63**	**.047**	**24.25**	**0.33**	**<.001**	**−0.36**	**0.18**	**.048**	**5.65**		
	Random slopes		− 4233.40	.293	24.26	0.33	<.001	−0.37	0.18	.050	5.77	1.41	−.17
Self esteem	Intercept only	.57	− 882.04		1.78	0.02	<.001				0.41		
*N* = 504	Fixed slopes		− 874.21	<.001	1.83	0.02	<.001	−0.05	0.01	<.001	0.41		
	Random slopes		**− 858.26**	**<.001**	**1.83**	**0.02**	**<.001**	**−0.05**	**0.01**	**<.001**	**0.41**	**0.17**	**−.08**
Wellbeing	Intercept only	.57	− 5100.03		41.98	0.56	<.001				11.02		
*N* = 498	Fixed slopes		− 5099.45	.283	41.64	0.64	<.001	0.36	0.34	.283	11.01		
	Random slopes		**− 5078.22**	**<.001**	**41.58**	**0.58**	**<.001**	**0.45**	**0.36**	**.218**	**9.74**	**3.92**	**.26**
Worry	Intercept only	.64	− 1113.28		1.61	0.03	<.001				0.53		
*N* = 504	Fixed slopes		− 1109.24	.004	1.57	0.03	<.001	0.03	0.01	.005	0.53		
	Random slopes		**− 1099.47**	**<.001**	**1.57**	**0.03**	**<.001**	**0.04**	**0.01**	**.007**	**0.57**	**0.19**	**−.29**
Other self-report measures		Model test			Fixed effects						Random effect		
		ICC	-2LL	*∆ p*	γ_00_	SE	*p*	γ_00_	SE	*p*	τ_00_	τ_11_	τ_01_
BAS Drive *N* = 501	Intercept only	.47	− 1205.31		1.20	0.02	<.001				0.46		
	Fixed slopes		**− 1200.16**	**.001**	**1.15**	**0.02**	**<.001**	**0.05**	**0.01**	**.001**	**0.46**		
	Random slopes		− 1197.56	.074	1.15	0.03	<.001	0.06	0.02	.002	0.51	0.17	−.37
BAS Fun *N* = 501	Intercept only	.51	**− 1123.33**		**1.70**	**0.02**	**<.001**				**0.46**		
	Fixed slopes		− 1122.51	.197	1.72	0.03	<.001	−0.02	0.02	.198	0.46		
	Random slopes		− 1121.00	.223	1.72	0.03	<.001	−0.02	0.02	.213	0.47	0.14	−.19
Cognitive restraint *N* = 502	Intercept only	.44	− 3695.66		13.46	0.16	g < .001				2.98		
	Fixed slopes		−3695.63	.813	13.44	0.19	<.001	0.02	0.11	.813	2.98		
	Random slopes		**− 3679.59**	**<.001**	**13.44**	**0.18**	**<.001**	**0.01**	**0.13**	**.933**	**3.09**	**1.67**	**−.22**
Emotional eating *N* = 502	Intercept only	.45	− 3038.18		5.81	0.10	<.001				1.83		
	Fixed slopes		− 3017.20	<.001	5.41	0.11	<.001	0.45	0.07	<.001	1.84		
	Random slopes		**− 3007.83**	**<.001**	**5.41**	**0.11**	**<.001**	**0.45**	**0.07**	**<.001**	**1.83**	**0.80**	**−.11**
Uncontrolled eating *N* = 501	Intercept only	.49	− 4004.05		20.47	0.22	<.001				4.10		
	Fixed slopes		− 3991.11	<.001	19.83	0.25	<.001	0.74	0.14	<.001	4.12		
	Random slopes		**− 3987.04**	**.017**	**19.82**	**0.26**	**<.001**	**0.76**	**0.15**	**<.001**	**4.52**	**1.56**	**−.36**
Lack of Perseverance *N* = 504	Intercept only	.42	− 967.906		2.11	0.02	<.001				0.35		
	Fixed slopes		**− 961.947**	**<.001**	**2.15**	**0.02**	**<.001**	**−0.05**	**0.01**	**<.001**	**0.35**		
	Random slopes		− 959.146	.061	2.15	0.02	<.001	−0.05	0.01	<.001	0.40	0.15	−.48
Lack of premeditation *N* = 504	Intercept only	.45	− 903.034		2.23	0.02	<.001				0.35		
	Fixed slopes		**− 889.513**	**<.001**	**2.29**	**0.02**	**<.001**	**−0.07**	**0.01**	**<.001**	**0.35**		
	Random slopes		− 888.698	.442	2.29	0.02	<.001	−0.07	0.02	<.001	0.37	0.09	−.36
Negative urgency *N* = 504	Intercept only	.54	− 1057.69		2.52	0.02	<.001				0.45		
	Fixed slopes		−1052.65	.001	2.48	0.03	<.001	0.05	0.01	.001	0.45		
	Random slopes		**−1046.61**	**.002**	**2.48**	**0.03**	**<.001**	**0.05**	**0.01**	**.003**	**0.46**	**0.16**	**−.17**
Sensation seeking *N* = 504	Intercept only	.73	− 1103.82		2.96	0.03	<.001				0.62		
	Fixed slopes		− 1102.97	.190	2.98	0.03	<.001	−0.02	0.01	.191	0.62		
	Random slopes		**− 1096.78**	**.002**	**2.98**	**0.03**	**<.001**	**−0.02**	**0.01**	**.172**	**0.64**	**0.17**	**−.19**
Negative life events *N* = 503	Intercept only	.48	− 3604.32		5.34	0.15	<.001				2.74		
	Fixed slopes		− 3602.05	.033	5.53	0.17	<.001	−0.21	0.10	.033	2.74		
	Random slopes		**− 3598.19**	**.021**	**5.53**	**0.18**	**<.001**	**−0.22**	**0.10**	**.030**	**3.09**	**0.64**	**−.65**
Risk involvement *N* = 503	Intercept only	.57	− 2803.55		4.19	0.09	<.001				1.80		
	Fixed slopes		− 2722.83	<.001	2.94	0.13	<.001	0.67	0.05	<.001	1.88		
	Random slopes		**− 2707.75**	**<.001**	**2.93**	**0.13**	**<.001**	**0.68**	**0.05**	**<.001**	**1.96**	**0.66**	**−.34**
Victimisation *N* = 503	Intercept only	.56	− 4294.59		9.96	0.29	<.001				5.61		
	Fixed slopes		− 4293.69	.182	10.16	0.32	<.001	−0.24	0.18	.182	5.61		
	Random slopes		**− 4289.18**	**.011**	**10.16**	**0.33**	**<.001**	**−0.24**	**0.19**	**.199**	**6.01**	**1.99**	**−.31**
Behavioural measures		Model test			Fixed effects						Random effects		
		ICC	-2LL	*∆ p*	γ_00_	SE	*p*	γ_10_	SE	*p*	τ_00_	τ_11_	τ_01_
Memory bias *N* = 504	Intercept only	.44	− 732.596		−0.39	0.02	<.001				0.30		
	Fixed slopes		− 689.730	<.001	−0.48	0.02	<.001	0.10	0.01	<.001	0.31		
	Random slopes		**− 686.670**	**.046**	**−0.48**	**0.02**	**<.001**	**0.10**	**0.01**	**<.001**	**0.33**	**0.11**	**−.31**
Non-social interpretation bias *N* = 504	Intercept only	.48	− 1794.36		−0.41	0.04	<.001				0.70		
	Fixed slopes		**− 1788.87**	**<.001**	**−0.34**	**0.04**	**<.001**	**−0.08**	**0.02**	**<.001**	**0.70**		
	Random slopes		− 1786.81	.128	−0.34	0.04	<.001	−0.08	0.02	.001	0.75	0.23	−.30
Social interpretation bias *N* = 504	Intercept only	.54	− 2021.78		0.61	0.05	<.001				0.91		
	Fixed slopes		−2018.65	.012	0.68	0.05	<.001	−0.07	0.03	.012	0.91		
	Random slopes		**−2005.68**	**<.001**	**0.68**	**0.05**	**<.001**	**−0.07**	**0.03**	**.023**	**0.97**	**0.41**	**−.30**

***Note***: Bold indicates best fitting model; only variables that showed high stability were modelled; *ICC* = Intraclass Correlation Coefficient from the intercept only model; −2LL = Log-likelihood value; *∆ p* = *p*-value for change in model fit; SE = Standard Error; γ_00_ = Intercept; γ_10_ = Slope; τ_00_ = Intercept variance; τ_11_ = Slope variance; τ_01_ = Covariance between random effects

Anxiety was best described by the random slopes model and there was no change in average scores across waves, γ_00_ = 13.56, *p* < .001, γ_10_ = 0.21, *p* = .283. Depression was best described by the random slopes model and there was an increase in scores from baseline, γ_00_ = 8.31, *p* < .001, γ_10_ = 0.94, *p* < .001. Rumination was best described by the random slopes model and there was an increase in scores from baseline, γ_00_ = 5.75, *p* < .001, γ_10_ = 0.39, *p* < .001. Resilience was best described by the fixed slopes model and there was no change observed across waves, γ_00_ = 24.25, *p* < .001, γ_10_ = − 0.36, *p* = .048. Self-esteem was best described by the random slopes model and there was a decrease in scores from baseline, γ_00_ = 1.83, *p* < .001, γ_10_ = − 0.05, *p* < .001. Wellbeing was best described by the random slopes model and there was no change in scores across waves, γ_00_ = 41.58, *p* < .001, γ_10_ = 0.45, *p* = .218. Worry was best described by the random slopes model and there was a marginal increase in scores from baseline, γ_00_ = 1.57, *p* < .001, γ_10_ = 0.04, *p* = .007. In sum, for the self-report mood variables that showed change, this was reflected by mood worsening across waves.

BAS Drive was best described by the fixed slopes model and there was an increase in average scores from baseline, γ_00_ = 1.15, *p* < .001, γ_10_ = 0.05, *p* = .001. BAS Fun was best described by the intercept only model, therefore no fixed or random effects were observed. Cognitive restraint was best described by the random slopes model and there was no change observed across waves, γ_00_ = 13.44, *p* < .001, γ_10_ = 0.01, *p* = .933. Emotional eating was best described by the random slopes model and there was an increase in scores from baseline, γ_00_ = 5.41, *p* < .001, γ_10_ = 0.45, *p* < .001. Uncontrolled eating was best described by the random slopes model and there was an increase in scores from baseline, γ_00_ = 19.82, *p* < .001, γ_10_ = 0.76, *p* < .001. Lack of perseverance was best described by the fixed slopes model and there was a decrease in scores from baseline, γ_00_ = 2.15, *p* < .001, γ_10_ = − 0.05, *p* < .001. Lack of premeditation was best described by the fixed slopes model and there was a decrease in scores from baseline, γ_00_ = 2.29, *p* < .001, γ_10_ = − 0.07, *p* < .001. Negative urgency was best described by the random slopes model and there was an increase in scores from baseline, γ_00_ = 2.48, *p* < .001, γ_10_ = 0.05, *p* = .003. Sensation seeking was best described by the random slopes model and there was no change observed across waves, γ_00_ = 2.98, *p* < .001, γ_10_ = − 0.02, *p* = .172. Negative life events was best described by the random slopes model and there was no change observed across waves, γ_00_ = 5.53, *p* < .001, γ_10_ = − 0.22, *p* = .030. Risk involvement was best described by the random slopes model and there was an increase in scores from baseline, γ_00_ = 2.93, *p* < .001, γ_10_ = 0.68, *p* < .001. Victimisation was best described by the random slopes model and there was no change observed across waves, γ_00_ = 10.16, *p* < .001, γ_10_ = − 0.24, *p* = .199.

Memory bias was best described by the random slopes model and there was an increase in average scores from baseline, γ_00_ = − 0.48, *p* < .001, γ_10_ = 0.10, *p* < .001, reflecting an increase in negative bias across waves. Non-social interpretation bias was best described by the fixed slopes model and there was a decrease in scores from baseline, γ_00_ = − 0.34, *p* < .001, γ_10_ = − 0.08, *p* < .001, reflecting a decrease in negative bias across waves. Social interpretation bias was best described by the random slopes model and there was no change in scores across waves, γ_00_ = 0.68, *p* < .001, γ_10_ = − 0.07, *p* = .023.

## Discussion

The current paper presents the CogBIAS-L-S cohort profile and examines stability and change in a wide range of psychological variables that were assessed across three waves of data collection. This study is one of the largest to track cognitive and emotional development across early to middle adolescence. Over 500 UK secondary school students participated in the study and completed repeated assessments at three waves, spaced approximately 12 to 18 months apart. A large proportion of the sample was retained, as none of the schools dropped out of the study. In total, we observed a 19% drop-out rate by Wave 3. The small amount of attrition was related to pupils either leaving the school, or being absent on the day of testing. Slightly more female participants were retained in the final sample. We observed substantial differential stability in our measures, as individual differences were largely maintained over time. Differential stability was greater for the self-report, compared to the behavioural measures, which could partly be explained by low measurement reliability reflected in some of the behavioural measures. We also observed adolescent-typical developmental changes, which were in line with our expectations, reflected by: (*i*) worsening mood outcomes, (*ii*) increasing impulsivity-related behaviour, and (*iii*) improvements in executive functions.

Reliability was assessed by examining the internal consistency of the measures, as lack of differential stability across waves could simply reflect poor measurement reliability. Across the self-report mood and other measures, internal consistency was very good. Only the Separation Anxiety subscale from the RCADS-SF [34] did not reach a high level. This was likely due to the fact that the scale was designed to assess anxiety as a total score, therefore the subscales may not contain enough items to reflect good levels of internal consistency. For the behavioural measures, internal consistency could only be examined for the Interpretation Bias variables (using McDonald’s *ω*), and the RT based measures, using split-half estimates. Unfortunately, internal consistency could not be examined for the count based measures, such as the BART and the Memory Bias task. Overall, internal consistency for the behavioural measures was low, as only the Negative Social variable from the AIBQ reached a high level. The Dot-Probe variables showed the lowest level of internal consistency, as estimates were non-significant in most cases. However, there was a trend for improvements in internal consistency across waves, which may have reflected improvements in attention control, which is characteristic of this period of development [11].

Differential stability in the measures was examined in order to assess the stability of individual differences across waves. High levels of stability were observed for all but one of the self-report mood measures. The Distraction subscale from the CRSS [39] did not show high stability across waves, suggesting that this particular construct is not stable across early to middle adolescence. The other self-report measures showed less stability than the mood measures, although individual differences were still largely maintained over time, as only four of the variables did not reach a high level. The variables that did not reach a high level of stability were: BIS, BAS-RR, Positive Life Events, and Pain Catastrophising. We did not expect Positive Life Events to be particularly stable, as it is a measure of life experiences, which are somewhat independent from individuals [69]. However, Negative Life Events did show high stability across waves, suggesting that individuals who experienced negative life events were likely to experience similar levels of negative life events in subsequent years. This could be explained by the experience of harsh and volatile family relationships, which would be likely to persist throughout adolescence.

For the behavioural measures, less stability was observed, as only Memory bias and the Interpretation bias variables reached a high level. The measures that were not stable included the BART (risk-taking), the Dot-Probe variables, Flanker Interference, and Food Approach Bias. In sum, we observed substantial differential stability in the self-report measures and less stability in the behavioural measures. It could be argued that the self-report variables are more trait based measures, while the behavioural variables are more state based, which could partly explain the lower stability observed for the behavioural measures. The instability of the behavioural measures could also have been more pronounced due to adolescent-typical developmental changes in the brain affecting cognitive functions, such as attention control and processing speed [2, 9, 10]. However, the RT based measures also showed poor internal consistency, calling into question the reliability of these tasks for assessing individual differences [31]. The Dot-probe variables showed no internal consistency and no differential stability, therefore future research should be very cautious about making inferences from this data.

In order to explore the data further, normative stability was examined with linear growth curve models for all continuous variables that showed high differential stability across three waves. For the self-report mood measures, we found that Depression, Rumination, and Worry increased across waves. Therefore, across the sample, these mood variables showed a worsening effect over time. We also found that Self-esteem decreased across waves, which reflected the same pattern of worsening mood over time. Although, Anxiety, Resilience and Wellbeing showed no significant change across waves. The decrease in mood that was observed supports previous research, as depression onset has been shown to peak at around 15 years of age [70]. While previous research suggests that anxiety onset typically occurs much earlier, in childhood and early adolescence [3]. This could explain why we did not observe any increase in anxiety, as our sample were around 13 years of age at Wave 1, therefore anxiety onset may have already peaked. Our results suggest that early adolescence may be a critical period for the delivery of mood interventions, which focus on decreasing depression, rumination and worry, as well as increasing self-esteem. The random slopes model provided the best fit to the data in most cases, showing that individual variability in change was substantial. Future research should attempt to explore this random growth, by conducting growth mixture models, which can identify different classes of individuals based on individual growth trajectories [30].

For the other self-report variables, we found a decrease in Lack of Perseverance and Lack of Premeditation across waves. We also found an increase in BAS Drive across waves. This could have been explained by the development of better executive functions, such as planning and goal-setting, which is typical during adolescence [10]. Yet, we found an increase in levels of impulsive behaviour, including Negative urgency, Emotional eating, Uncontrolled eating, and Risk involvement. This also reflects adolescent-typical behaviour, such as the dual-systems model, which proposes that protracted neural development in the prefrontal cortex contributes to increasing levels of risk-taking during adolescence [7–9]. Substantial individual variability in change was observed, as most variables were best explained by the random slopes model, which could justify further growth mixture analyses. However, Lack of Premeditation, Lack of Perseverance, and BAS Drive, were best explained by the fixed slopes model, suggesting that most of the sample changed in the same direction for these variables.

For the behavioural measures, we found an increase in Memory Bias, as participants developed more of a negative memory bias across waves. This reflected the same pattern as the self-report mood measures, which showed a worsening mood effect over time. However, we found a decrease in Non-social Interpretation Bias, as participants developed less of a negative bias over time. We found no significant change in Social Interpretation Bias across waves. To our knowledge, no previous studies have examined the longitudinal development of cognitive biases in normative adolescent samples. Yet, previous research has suggested that memory bias is a construct that is particularly relevant to depression, while interpretation bias is relevant to both anxiety and depression [71]. This could partly explain why we found an increase in memory bias, but not for interpretation bias. Although beyond the scope of the current paper, these findings could be explored further, by examining the co-development of cognitive biases and symptoms of anxiety and depression, to understand these pathways better.

### Strengths and limitations

To our knowledge, CogBIAS-L-S is the largest study of its kind, investigating the development of cognitive biases in relation to emotional vulnerability and resilience in a normative adolescent sample. A wide range of measures were assessed, capturing multiple aspects of psychological functioning, with a primary focus on mood variables. The study did not rely solely on self-report measures, as a range of behavioural measures were also assessed, which could highlight potential mechanisms underlying mental health outcomes. We tested and retained a large sample across three waves and found substantial variability in the measures, which will allow for further analyses on the complex interplay between measures over time. For example, much variability in anxiety and depressive symptoms was observed, with a substantial proportion (around 20%) of the sample reaching clinical levels, based on previous cut-off scores [72]. Therefore, CogBIAS-L-S provides a rich source of data, which has the potential to advance current knowledge of adolescent psychological development, particularly that of cognitive biases.

Some limitations of the study should be noted. While the inclusion of behavioural measures was a strength of the study, unfortunately many of these showed low reliability. For example, the Dot-probe variables showed no internal consistency (i.e., non-significant), as well no differential stability, which was likely due to lack of measurement reliability. Therefore, making inferences about attentional biases will be problematic in future studies. This finding has been reported in the literature recently, therefore future researchers should consider using alternative measures of attentional bias, or find ways to improve the reliability of the Dot-probe task [73]. We advocate the reporting of reliability in all studies using behavioural measures, in the same way that reliability is reported for self-report measures, as this will advance and improve measures going forward [74]. The other behavioural measures did show some degree of internal consistency and differential stability, albeit less than the self-report measures. Consistent reporting of behavioural task reliability in future studies will allow researchers to develop criteria for judging adequate levels of reliability, which may not be comparable to self-report measures.

Data was collected in a group setting, which may have led to distraction or even demand characteristics, due to participants sitting next to their peers. Although, measures were taken to reduce this possibility. Sessions were conducted in exam conditions and participants were instructed not to talk or look at their peer’s computer screens. The test sessions were quite long and included a lot of measures, therefore fatigue may have been experienced by some participants. Not all participants were able to finish the test battery for this reason. We also experienced some IT issues, which resulted in missing data at various stages of the assessment battery, although attempts were made to recover as much missing data as possible.

## Conclusions

CogBIAS-L-S represents a three-wave longitudinal study investigating psychological development across early to middle adolescence. A wide range of psychological variables were assessed, including many mood and impulsivity-related self-report and cognitive behavioural measures. Substantial differential stability was observed, as individual differences were largely maintained across waves, in line with classical test theory. This was especially true for the self-report measures, in comparison to the behavioural measures, which showed lower stability across waves. The substantial differential stability observed suggests that many mood and impulsivity-related behaviours show onset in early adolescence. This highlights a potential intervention window at the beginning of secondary school, before psychological characteristics become particularly stable. Some sample level normative changes were observed across waves. We found a pattern reflecting worsening mood (e.g., increasing levels of depression and rumination), increasing impulsivity-related behaviour (e.g., risk-taking and uncontrolled eating), as well as improvements in executive functions (e.g., planning and control). Future studies will investigate predictive associations between the variables, as well as combining analyses with the genome-wide data that has been collected. It is hoped that our results will advance the literature on risk and protective pathways during adolescence. Beyond that, it has the potential to contribute to the development of new interventions designed to improve mood and impulsivity-related outcomes for adolescents.

## Data Availability

The datasets used and/or analysed during the current study will be made available from the Principal Investigator, Professor Elaine Fox, upon reasonable request.
